# Retraction: The comparative effectiveness of 55 interventions in obese patients with polycystic ovary syndrome: A network meta-analysis of 101 randomized trials

**DOI:** 10.1371/journal.pone.0327327

**Published:** 2025-07-09

**Authors:** The PLOS One Editors

The *PLOS One* Editors retract this article [1] due to concerns about:

- Reliance on retracted work cited in References 20, 34, 35, 55, 59, 66, 77, and 98.
- The reliability of the reported results and conclusions.

MAM, AM, EAH, MA, ME, and AM did not agree with the retraction. MIA, EM, and OO either did not respond directly or could not be reached.
